# Clinical characteristics and outcomes of acute kidney injury in patients with severe fever with thrombocytopenia syndrome

**DOI:** 10.3389/fmicb.2023.1236091

**Published:** 2023-09-13

**Authors:** Zhongwei Zhang, Xue Hu, Qunqun Jiang, Wenjia Hu, Anling Li, Liping Deng, Yong Xiong

**Affiliations:** ^1^Department of Infectious Disease, Zhongnan Hospital of Wuhan University, Wuhan, China; ^2^Department and Institute of Infectious Disease, Tongji Hospital, Tongji Medical College, Huazhong University of Science and Technology, Wuhan, China; ^3^Department of Clinical Laboratory, Center for Gene Diagnosis, and Program of Clinical Laboratory Medicine, Zhongnan Hospital of Wuhan University, Wuhan, China

**Keywords:** severe fever with thrombocytopenia syndrome, acute kidney injury, clinical characteristics, risk factor, mortality

## Abstract

**Background:**

Severe fever with thrombocytopenia syndrome (SFTS) is an emerging zoonosis caused by a novel bunyavirus. Until recently, the SFTS related acute kidney injury (AKI) was largely unexplored. This study aimed to investigate the clinical characteristics and outcomes of AKI in patients with SFTS.

**Methods:**

The non-AKI and AKI groups were compared in terms of general characteristics, clinical features, laboratory parameters and cumulative survival rate. The independent risk factors for in-hospital mortality in patients with SFTS were analyzed by multivariate logistic regression to identify the population with poor prognosis.

**Results:**

A total of 208 consecutive patients diagnosed with SFTS were enrolled, including 153 (73.6%) patients in the non-AKI group and 55 (26.4%) patients in the AKI group. Compared with patients without AKI, patients with AKI were older and had a higher frequency of diabetes. Among these laboratory parameters, platelet count, albumin and fibrinogen levels of patients with AKI were identified to be significantly lower than those of patients without AKI, while ALT, AST, ALP, triglyceride, LDH, BUN, uric acid, creatine, Cys-C, β2-MG, potassium, AMY, lipase, CK-MB, TnI, BNP, APTT, thrombin time, D-dimer, CRP, IL-6, PCT and ESR levels were significantly higher in patients with AKI. A higher SFTS viral load was also detected in the AKI patients than in the non-AKI patients. The cumulative survival rates of patients at AKI stage 2 or 3 were significantly lower than those of patients without AKI or at AKI stage 1. However, there was no significant difference in the cumulative survival rates between patients without AKI and those with stage 1 AKI. Univariate and multivariate binary logistic regression analyses demonstrated that stage 2 or 3 AKI was an independent risk factor for in-hospital mortality in patients with SFTS.

**Conclusion:**

AKI is associated with poor outcomes in patients with SFTS, especially patients at AKI stage 2 or 3, who generally have high mortality. Our findings support the importance of early identification and timely treatment of AKI in patients with SFTS.

## Introduction

Severe fever with thrombocytopenia syndrome (SFTS) is an emerging infectious disease caused by a novel bunyavirus that was reported in rural areas of China in 2009 (Yu et al., 2011). SFTS is mainly transmitted by contact with ticks, and can be transmitted among humans (Bao et al., 2011), even direct infection of SFTS virus from cats and dogs to humans has been reported, which is recognized as a social problem (Park et al., 2019; Yamanaka et al., 2020). Patients with mild SFTS can present with fever, thrombocytopenia, anorexia, dizziness, headache, nausea, vomiting and diarrhea, while patients with serious SFTS might develop multiple organ dysfunction syndrome including encephalopathy, cardiac failure, acute respiratory distress syndrome, severe pancreatitis, acute kidney injury (AKI), and disseminated intravascular coagulation, with a reported mortality rate ranging from 2.5% to 30% in various epidemic areas (Zhang et al., 2012; Liu S. et al., 2014; Li et al., 2018). The extensive distribution, and high morbidity and mortality of SFTS make it a significant newly emerging infectious disease in China.

Critically ill patients with SFTS always have a high mortality rate, and the clinical manifestations and laboratory parameters could be used to predict the outcomes of patients with SFTS, which have been explored with high interest (Wen et al., 2014; Xu et al., 2018). Among these definite predictors, elevated serum creatinine (sCr) levels have been frequently demonstrated to be closely related to poor prognosis (Cui et al., 2015; Nie et al., 2020; He et al., 2021). The elevated sCr levels in a short time typically represent AKI. It suggests that AKI may be closely related to the adverse outcomes of patients with SFTS. Hence, a comprehensive understanding of the clinical characterization of AKI and its associated outcomes is crucial for formulating personalized treatment strategies to improve the prognosis of patients with SFTS. However, until recently, the data for AKI in SFTS were limited. We performed a study to investigate the incidence, clinical manifestations and outcomes of AKI in a retrospective cohort of hospitalized patients with SFTS.

## Methods

### Patients

A total of 208 consecutive patients with SFTS admitted to the Department of Infectious Disease, Zhongnan Hospital of Wuhan University between August 2016 and June 2023 were included in a retrospective cohort. The patients were then divided into the AKI (*n* = 153) and non-AKI groups (*n* = 55) according to the presence of AKI. According to the current Kidney Disease: Improving Global Outcomes (KDIGO) staging criteria for AKI, patients in the AKI group were further divided into three subgroups: stage 1 AKI group (*n* = 23), stage 2 AKI group (*n* = 17) and stage 3 AKI group (*n* = 15).

The criteria of diagnosing SFTS were as follows (Liu Q. et al., 2014): (1) acute fever (temperature >37.3°C for over 24 h) with thrombocytopenia; (2) laboratory-confirmed SFTS virus infection by detection of viral RNA through polymerase chain reaction assay. Patients were excluded if they fulfilled one or more of the following criteria: (1) age ≤18 years or ≥80 years, (2) reduced glomerular filtration rate or chronic kidney disease had been confirmed before admission, (3) previous kidney transplantation; (4) the presence of preterminal comorbidities (heart disease New York Heart Association III–IV, severe chronic obstructive pulmonary disease), (5) any other types of immunodeficiency. (6) history of malignant tumor.

Diagnosis of AKI was based on the KDIGO guidelines (Palevsky et al., 2013): (1) increase in sCr by ≥26.5 μmol/L (or ≥50%) within 48 h; or (2) increase in sCr to ≥1.5 times the baseline value, which is known or presumed to have within the previous 7 days; or (3) urine volume <0.5 mL/kg/h for 6 h. The KDIGO criteria for AKI staging were as follows: (1) AKI stage 1, increase in sCr by 1.5–1.9 times the baseline value, or increase in sCr by 26.5 μmol/L within 48 h, or urine volume <0.5 mL/kg/h and >0.3 mL/kg/h for 6–12 h; (2) AKI stage 2, increase in sCr by 2.0–2.9 times the baseline value, or urine volume <0.5 mL/kg/h and >0.3 mL/kg/h for ≥12 h; (3) AKI stage 3, increase in sCr by 3.0 times the baseline value, or increase in sCr to 353.6 μmol/L, or start of renal replacement therapy, or urine volume <0.3 mL/kg/h for ≥24 h.

During hospitalization, all patients received routine supportive treatment, including bed rest, adequate nutritional support, intensive care and monitoring. Complications including coagulation disorders, thrombocytopenia crisis, neutropenia, acute respiratory distress syndrome, septic shock and AKI were closely monitored and managed immediately.

### Data collection

The medical records of patients with SFTS were reviewed, demographic details, comorbid conditions, symptoms, signs and laboratory tests data including white blood cell (WBC) count and percentage, neutrophil count and percentage, lymphocyte count and percentage, hemoglobin, platelet (PLT) count, alanine aminotransferase (ALT), aspartate aminotransferase (AST), total bilirubin (TBIL), albumin, alkaline phosphatase (ALP), gamma glutamyl transpeptidase (GGT), lactate dehydrogenase (LDH), total cholesterol (TC), triglyceride (TG), blood urea nitrogen (BUN), uric acid, sCr, cystatin C (Cys-C), β2-microglobulin (β2-MG), sodium, potassium, calcium, magnesium, chlorine, phosphorus, amylase (AMY), lipase, creatinine kinase (CK), creatinine kinase myocardial B fraction (CK-MB), troponin I (TnI), brain natriuretic peptide (BNP), prothrombin time (PT), international normalized ratio (INR), prothrombin activity (PTA), activated partial thromboplastin time (APTT), thrombin time (TT), fibrinogen, D-dimer, C-reactive protein (CRP), procalcitonin (PCT), interleukin-6 (IL-6), erythrocyte sedimentation rate (ESR), SFTS viral load, fecal occult blood test (OBT), urine protein test (UPT), urine occult blood test (UOBT) and survival time were collected at the time of AKI diagnosis.

### Statistical analysis

All data were analyzed with IBM SPSS statistical analysis software (version 23.0, Chicago, United States), and *p* < 0.05 (two-sided) was considered statistically significant. Categorical variables were shown as numbers (percentages) and were compared by the Chi-square test or Fisher’s exact test. Continuous variables were shown as the means ± standard deviations for data with a normal distribution or medians with interquartile ranges (P25–P75) for data with a non-normal distribution, which were compared by the student’s *t*-test or Mann–Whitney *U* test, respectively. Correlations between numerical laboratory parameters were analyzed using Spearman correlation analysis. The cumulative survival rates of patients were evaluated using the Kaplan–Meier method and were compared by the log-rank test. Logistic regression analysis was conducted to identify the risk factors for fatal outcome of patients. All clinical data including demographic, comorbid conditions, clinical symptoms and laboratory indicators were included for univariate analysis, from which biologically plausible variables with *p-*value <0.05 were entered into the multivariate model by a stepwise method.

## Results

### Demographic and clinical manifestations

A total of 208 consecutive patients diagnosed with SFTS were enrolled, including 153 patients in the non-AKI group and 55 patients in the AKI group. Among these patients, 48 patients were diagnosed with AKI at admission, and seven patients were diagnosed with AKI during hospitalization. The time between symptom onset and diagnosis of AKI was 8 (7–12) days. Compared with patients without AKI, patients with AKI were older, and had a higher frequency of diabetes and more days from onset to admission. No difference was observed in the frequency of sex or hypertension between the two groups. Among all these clinical manifestations, headache, anorexia, abdominal pain, diarrhea, petechia, encephalopathy and hepatosplenomegaly were significantly overrepresented in patients in the AKI group compared with patients in the non-AKI group. However, there was no significant difference in other clinical features including dizziness, cough, sputum, chest distress, nausea, vomiting and the frequency of fever >38°C (Table 1).

**Table 1:** Comparison of demographics, comorbid conditions and clinical symptoms between the non-AKI and AKI groups.

	All (*n* = 208)	Non-AKI (*n* = 153)	AKI (*n* = 55)	*p*-value
Male, *n* (%)	110 (52.9)	81 (52.9)	29 (52.7)	0.978
Age (years)	65 ± 8	64 ± 8	67 ± 7	0.024
Diabetes, *n* (%)	15 (7.2)	7 (4.6)	8 (14.5)	0.028
Hypertension, *n* (%)	53 (25.5)	36 (23.5)	17 (30.9)	0.281
Days from onset to admission	7 (6–9)	6 (5–7)	8 (7–10)	0.033
*Clinical manifestations, n (%)*				
Fever >38°C	50 (24.0)	33 (21.6)	17 (30.9)	0.164
Headache	40 (19.5)	24 (15.7)	16 (29.1)	0.031
Dizziness	62 (29.8)	43 (28.1)	19 (34.5)	0.370
Cough	55 (26.4)	39 (25.5)	16 (29.1)	0.604
Sputum	41 (19.7)	29 (19.0)	12 (21.8)	0.647
Chest distress	42 (20.2)	28 (18.3)	14 (25.5)	0.257
Anorexia	153 (73.6)	106 (69.3)	47 (85.5)	0.020
Nausea	160 (76.9)	116 (75.8)	44 (80.0)	0.528
Vomiting	53 (25.5)	41 (26.8)	12 (21.8)	0.467
Abdominal pain	55 (26.4)	34 (22.2)	21 (38.2)	0.021
Diarrhea	33 (15.9)	20 (13.1)	13 (23.6)	0.012
Petechia	22 (10.6)	10 (6.5)	12 (21.8)	0.032
Encephalopathy	32 (15.4)	14 (9.2)	18 (32.7)	<0.001
Hepatosplenomegaly	23 (11.1)	12 (7.8)	11 (20.0)	0.014

### Laboratory test results

Among these laboratory parameters, PLT count, albumin and fibrinogen levels of patients with AKI were identified to be significantly lower than those of patients without AKI, while ALT, AST, ALP, LDH, TG, BUN, uric acid, sCr, Cys-C, β2-MG, potassium, phosphorus, AMY, lipase, CK-MB, TnI, BNP, APTT, TT, D-dimer, CRP, IL-6, PCT and ESR levels were significantly higher in patients with AKI. A higher SFTS viral load was also detected in the AKI patients than in the non-AKI patients. In terms of routine urine test examinations, the urine RBC count in the AKI patients was significantly higher than that in the non-AKI patients. No significant difference was observed for WBC, neutrophil and lymphocyte counts, TBIL, TC, sodium, PT, INR, APTT or frequency of OBT positivity between the two groups (Table 2).

**Table 2:** Comparison of laboratory parameters in the two study groups.

Variables	Normal range	All (*n* = 208)	Non-AKI (*n* = 153)	AKI (*n* = 55)	*p*-value
WBC (10^9^/L)	3.5–9.5	4.1 (2.3–6.7)	4.0 (2.1–6.9)	4.2 (2.6–6.4)	0.778
Neutrophil (%)	40.0–75.0	70.1 (54.0–82.4)	68.9 (51.6–82.3)	72.7 (60.5–82.9)	0.160
Neutrophil (10^9^ /L)	1.8–6.3	2.7 (1.2–5.1)	2.6 (1.1–5.0)	3.1 (1.5–5.3)	0.375
Lymphocyte (%)	20.0–50.0	20.8 (11.6–31.2)	20.6 (11.4–32.6)	20.9 (12.4–27.5)	0.590
Lymphocyte (10^9^/L)	1.1–3.2	0.7 (0.5–1.1)	0.7 (0.5–1.1)	0.7 (0.5–1.1)	0.511
Hemoglobin (g/L)	130–175	122 ± 23	124 ± 23	118 ± 24	0.140
PLT (10^9^/L)	125–350	41 (28–57)	42 (31–57)	34 (22–58)	0.050
ALT (U/L)	9–50	74 (45–130)	69 (45–113)	92 (48–204)	0.022
AST (U/L)	15–40	187 (91–419)	152 (85–315)	402 (134–825)	<0.001
TBIL (μmol/L)	5–21	11.7 (8.6–17.2)	10.8 (8.3–16.1)	13.7 (9.4–20.8)	0.084
Albumin (g/L)	40–55	29.9 ± 5.1	30.5 ± 4.7	28.0 ± 5.6	0.001
ALP (U/L)	30–120	74 (58–103)	71 (55–88)	106 (65–159)	<0.001
GGT (U/L)	8–57	40 (24–97)	33 (22–79)	48 (32–128)	<0.001
LDH (U/L)	125–243	708 (402–1,000)	634 (391–961)	925 (562–1,622)	0.002
TC (mmol/L)	0–5.18	2.94 (2.41–3.57)	2.91 (2.40–3.54)	3.07 (2.42–3.66)	0.459
TG (mmol/L)	0–1.70	2.23 (1.53–3.05)	2.01 (1.49–2.94)	2.65 (1.96–3.52)	0.026
BUN (mmol/L)	2.8–7.6	5.3 (4.0–8.0)	4.9 (3.6–6.1)	8.5 (6.2–15.8)	<0.001
Uric acid (μmol/L)	119–417	305 (243–397)	270 (227–326)	444 (387–513)	<0.001
sCr (μmol/L)	64–104	75 (64–110)	69 (61–79)	197 (146–299)	<0.001
Cys-C (mg/L)	0–1.2	1.26 (1.01–1.58)	1.16 (0.95–1.43)	1.68 (1.37–2.75)	<0.001
β2-MG (μg/L)	1,000–3,000	4,067 (3422–5,410)	3,616 (3073–4,210)	5,432 (4565–14,821)	<0.001
Sodium (mmol/L)	137–147	135 (132–138)	135 (132–138)	134 (131–139)	0.868
Potassium (mmol/L)	3.5–5.3	3.6 (3.3–4.2)	3.5 (3.2–3.9)	4.0 (3.6–4.5)	<0.001
Calcium (mmol/L)	2.0–2.7	1.93 (1.82–2.03)	1.93 (1.88–2.02)	1.95 (1.76–2.06)	0.425
Magnesium (mmol/L)	0.85–1.15	0.93 (0.81–1.09)	0.93 (0.81–1.03)	0.95 (0.80–1.15)	0.249
Chlorine (mmol/L)	96–106	103.0 (99.0–105.1)	103.2 (98.9–105.4)	102.3 (99.3–104.3)	0.320
Phosphorus (mmol/L)	0.8–1.5	1.09 (0.87–1.43)	0.98 (0.82–1.17)	1.52 (1.40–1.60)	<0.001
AMY (U/L)	0–90	146 (94–227)	130 (85–204)	192 (134–318)	<0.001
Lipase (U/L)	0–70	164 (78–315)	129 (67–260)	266 (117–465)	0.001
CK (U/L)	0–171	444 (147–1,197)	429 (132–997)	512 (182–1,582)	0.156
CK-MB (U/L)	0–25	32 (15–49)	28 (14–45)	39 (22–89)	0.004
TnI (pg/mL)	0–26.2	96.6 (34.7–218.5)	72.1 (40.9–207.0)	126.9 (75.8–432.5)	0.012
BNP (pg/mL)	0–1800	78.0 (128.3–192.5)	73.7 (58.4–120.5)	142.7 (123.5–280.8)	0.034
PT (s)	9.4–12.5	11.4 (10.7–12.0)	11.3 (10.6–12.0)	11.7 (11.1–12.7)	0.111
INR	0.85–1.15	1.04 (0.98–1.10)	1.03 (0.97–1.10)	1.06 (1.01–1.14)	0.107
PTA (%)	80–130	98 (88–109)	100 (90–109)	95 (85–108)	0.126
APTT (s)	25.1–36.5	42.1 (36.2–49.0)	41.0 (35.7–45.4)	48.0 (39.6–57.4)	<0.001
TT (s)	10.3–16.6	18.3 (16.6–22.3)	17.3 (16.1–19.9)	20.7 (17.1–24.7)	0.001
Fibrinogen (mg/dL)	238–498	237 (185–290)	249 (198–295)	223 (147–280)	0.017
D-dimer (ng/mL)	0–500	1,063 (472–2,230)	900 (403–1913)	1810 (612–3,340)	0.003
CRP (mg/L)	0–10.0	7.1 (2.4–17.5)	4.9 (1.6–14.9)	10.7 (6.4–28.0)	0.033
PCT (ng/mL)	0–0.05	0.26 (0.08–0.72)	0.18 (0.06–0.40)	0.89 (0.34–2.42)	<0.001
IL-6 (pg/mL)	0–7	41.0 (15.1–115.3)	33.3 (13.1–86.9)	113.4 (26.7–557.6)	<0.001
ESR (mm/h)	0–20	8 (4–14)	7 (4–12)	10 (7–23)	0.038
Urine RBC count	0–13.1	19.3 (8.5–65.4)	16.7 (7.4–40.9)	33.9 (14.1–146.0)	0.009
Viral load (log_10_ copies/mL)		4.2 (3.2–5.4)	4.0 (3.1–4.5)	5.5 (3.8–6.9)	0.002
OBT positive, *n* (%)		49 (23.6)	31 (20.3)	18 (32.8)	0.062
UPT positive, *n* (%)		171 (82.2)	125 (81.6)	46 (83.6)	0.747
UOBT positive, *n* (%)		163 (78.4)	118 (77.1)	45 (81.8)	0.468

**Figure 1:**
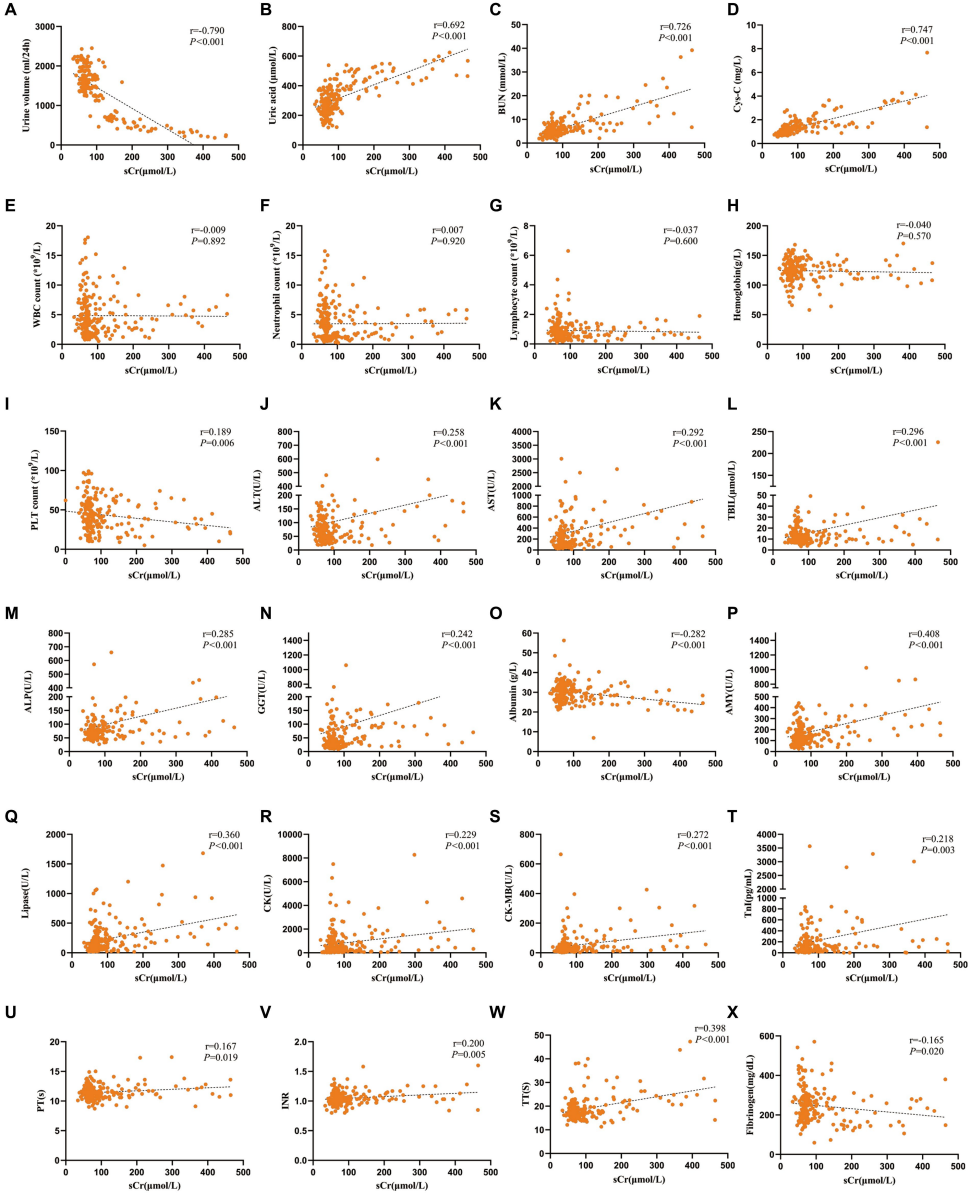
Spearman correlation analysis was carried out to analyze the relationship between the levels of sCr and other laboratory parameters in patients with SFTS. **(A)** Correlation analysis between sCr and urine volume. **(B)** Correlation analysis between sCr and uric acid. **(C)** Correlation analysis between sCr and BUN. **(D)** Correlation analysis between sCr and Cys-C. **(E)** Correlation analysis between sCr and WBC count. **(F)** Correlation analysis between sCr and neutrophil count. **(G)** Correlation analysis between sCr and lymphocyte count. **(H)** Correlation analysis between sCr and hemoglobin. **(I)** Correlation analysis between sCr and PLT count. **(J)** Correlation analysis between sCr and ALT. **(K)** Correlation analysis between sCr and AST. **(L)** Correlation analysis between sCr and TBIL. **(M)** Correlation analysis between sCr and ALP. **(N)** Correlation analysis between sCr and GGT. **(O)** Correlation analysis between sCr and albumin. **(P)** Correlation analysis between sCr and AMY. **(Q)** Correlation analysis between sCr and lipase. **(R)** Correlation analysis between sCr and CK. **(S)** Correlation analysis between sCr and CK-MB. **(T)** Correlation analysis between sCr and TnI. **(U)** Correlation analysis between sCr and PT. **(V)** Correlation analysis between sCr and INR. **(W)** Correlation analysis between sCr and TT. **(X)** Correlation analysis between sCr and Fibrinogen.

Spearman correlation analysis was conducted to analyze the correlation between sCr and other laboratory parameters. The results indicated that sCr was positively correlated with ALT, AST, TBIL, GGT, ALP, BUN, uric acid, Cys-C, AMY, lipase, CK, CK-MB, TnI, PT, INR, APTT and TT, but was negatively correlated with PLT count, albumin, urine volume and fibrinogen (Figure 1).

### Prognosis classification of patients with SFTS

**Figure 2:**
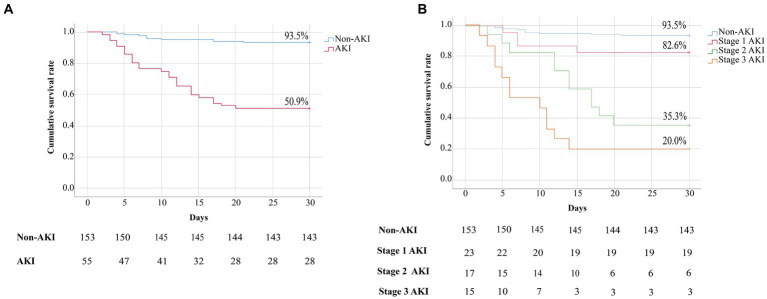
**(A)** Kaplan–Meier curves showing the cumulative survival rates of patients in the non-AKI and AKI groups. Comparison of the survival estimates was performed using the log rank test, non-AKI group vs. AKI group, *p* < 0.001. **(B)** Kaplan–Meier curves showing the cumulative survival rates of patients with SFTS based on AKI stage. Comparison of the survival estimates was performed using the log rank test, non-AKI group vs. stage 1 AKI group, *p* = 0.481; non-AKI group vs. stage 2 AKI or stage 3 AKI group, all *p* < 0.001; stage 1 AKI group vs. stage 2 AKI or stage 3 AKI group, *p* = 0.003, *p* < 0.001, respectively.

As shown in Figure 2A, the cumulative survival rate of patients in the AKI group was significantly lower than that in the non-AKI group (93.4% vs. 50.9%, *p* < 0.001). As shown in Figure 2B, patients in the AKI group were divided into three subgroups, and the cumulative survival rates of patients at AKI stages 1, 2 and 3 were 82.6%, 35.3% and 20.0%, respectively. Both the cumulative survival rates of patients at AKI stage 2 or 3 were significantly lower than those of patients without AKI (all *p* < 0.001). However, there was no significant difference in the cumulative survival rates between the stage 1 AKI and non-AKI groups (*p* = 0.066). Moreover, compared with patients at AKI stage 1, patients at AKI stage 2 or 3 also had significantly lower cumulative survival rates (*p* = 0.003, *p* < 0.001, respectively). Moreover, the clinical features and laboratory parameters of patients at AKI stages 1, 2 and 3 were compared. We revealed that patients with stage 2 or 3 AKI had more days from onset to admission, and higher serum levels of ALT, AST, LDH, uric acid, Cys-C, β2-MG, AMY, lipase, CK-MB, TnI, BNP, D-dimer, CRP, PCT, IL-6, ESR, viral load and urine RBC count than patients with stage 1 AKI (Supplementary Table S1).

### Risk factors for fatal outcomes

Univariable logistic regression analyses revealed that the presence of petechia, encephalopathy, hepatosplenomegaly and stage 2 or 3 AKI, old age, and decreased albumin, low fibrinogen, and elevated levels of AST, ALP, GGT, LDH, TG, uric acid, AMY, lipase, CK-MB, PT, APTT, TT, IL-6 and viral load were risk factors for fatal outcomes in patients with SFTS. The above variables were used for multivariable logistic regression analyses. It demonstrated that stage 2 or 3 AKI (OR, 10.329; 95% CI, 4.815–21.244), encephalopathy (OR, 5.935; 95% CI, 3.056–13.903), elevated levels of LDH (OR, 1.002; 95% CI, 1.001–1.003) and viral load (OR, 2.415; 95% CI, 1.702–4.026) were independent risk factors for mortality in patients with SFTS (Table 3).

**Table 3:** Univariable and multivariable logistic regression analyses of in-hospital mortality in patients with SFTS.

	Univariable analysis		Multivariable analysis	
	OR (95% CI)	*p*-value	OR (95% CI)	*p*-value
Male	1.436 (0.704–2.931)	0.320		
Age	1.057 (1.008–1.108)	0.022		
Diabetes	2.516 (0.806–7.855)	0.112		
Hypertension	1.012 (0.494–2.462)	0.812		
Days from onset to admission	1.036 (0.921–1.164)	0.560		
Fever >38°C	2.649 (0.946–5.632)	0.052		
Headache	2.072 (0.921–4.658)	0.078		
Anorexia	2.075 (0.815–5.285)	0.126		
Abdominal pain	1.429 (0.661–3.086)	0.364		
Diarrhea	1.301 (0.517–3.274)	0.576		
Petechia	3.094 (1.191–8.037)	0.020		
Encephalopathy	7.381 (3.220–16.917)	<0.001	5.935 (3.056–13.903)	0.012
Hepatosplenomegaly	4.501 (1.794–11.294)	0.001		
Stage 2 or 3 AKI	12.536 (8.454–23.178)	<0.001	10.329 (4.815–21.244)	<0.001
WBC (10^9^/L)	1.014 (0.916–1.122)	0.787		
Neutrophil (%)	0.999 (0.992–1.007)	0.831		
Neutrophil (10^9^ /L)	0.999 (0.885–1.127)	0.987		
Lymphocyte (%)	0.986 (0.959–1.014)	0.311		
Lymphocyte (10^9^/L)	1.120 (0.711–1.762)	0.625		
Hemoglobin (g/L)	0.991 (0.977–1.005)	0.206		
PLT (10^9^/L)	0.982 (0.964–1.001)	0.059		
ALT (U/L)	1.002 (0.999–1.004)	0.131		
AST (U/L)	1.002 (1.001–1.003)	<0.001		
TBIL (μmol/L)	1.016 (0.998–1.034)	0.077		
Albumin (g/L)	0.903 (0.834–0.977)	0.011		
ALP (U/L)	1.006 (1.002–1.009)	0.002		
GGT (U/L)	1.003 (1.001–1.005)	0.008		
LDH (U/L)	1.003 (1.001–1.004)	<0.001	1.002 (1.001–1.003)	0.006
TC (mmol/L)	0.712 (0.410–1.239)	0.230		
TG (mmol/L)	1.276 (1.000–1.628)	0.050		
BUN (mmol/L)	1.000 (0.990–1.010)	0.816		
Uric acid (μmol/L)	1.009 (1.005–1.012)	<0.001		
Cys-C (mg/L)	1.132 (0.931–1.375)	0.214		
Sodium (mmol/L)	0.984 (0.926–1.046)	0.604		
Potassium (mmol/L)	4.349 (2.282–8.290)	<0.001		
AMY (U/L)	1.002 (1.001–1.003)	0.002		
Lipase (U/L)	1.002 (1.000–1.003)	0.007		
CK (U/L)	1.000 (1.000–1.000)	0.040		
CK-MB (U/L)	1.009 (1.004–1.014)	<0.001		
TnI (pg/mL)	1.001 (1.000–1.001)	0.069		
PT (s)	1.474 (1.118–1.944)	0.006		
INR	1.003 (0.990–1.017)	0.627		
PTA (%)	0.984 (0.963–1.005)	0.140		
APTT (s)	1.073 (1.039–1.108)	<0.001		
TT (s)	1.075 (1.022–1.131)	0.005		
Fibrinogen (mg/dL)	0.988 (0.982–0.995)	<0.001		
D-dimer (ng/mL)	1.001 (1.000–1.003)	0.397		
CRP (mg/L)	1.025 (0.983–1.069)	0.254		
PCT (ng/mL)	1.118 (0.981–1.274)	0.096		
IL-6 (pg/mL)	1.008 (1.002–1.014)	0.012		
ESR (mm/h)	1.025 (0.996–1.056)	0.096		
Viral load	3.309 (1.855–5.902)	<0.001	2.415 (1.702–4.026)	0.026
OBT positive	1.769 (0.794–3.940)	0.163		
UPT positive	1.144 (0.439–2.978)	0.783		
UOBT positive	1.629 (0.635–4.180)	0.310		
Urine RBC count	1.000 (1.000–1.001)	0.692		

### Longitudinal changes in laboratory parameters in patients without AKI or at AKI stages 1, 2 and 3

**Figure 3:**
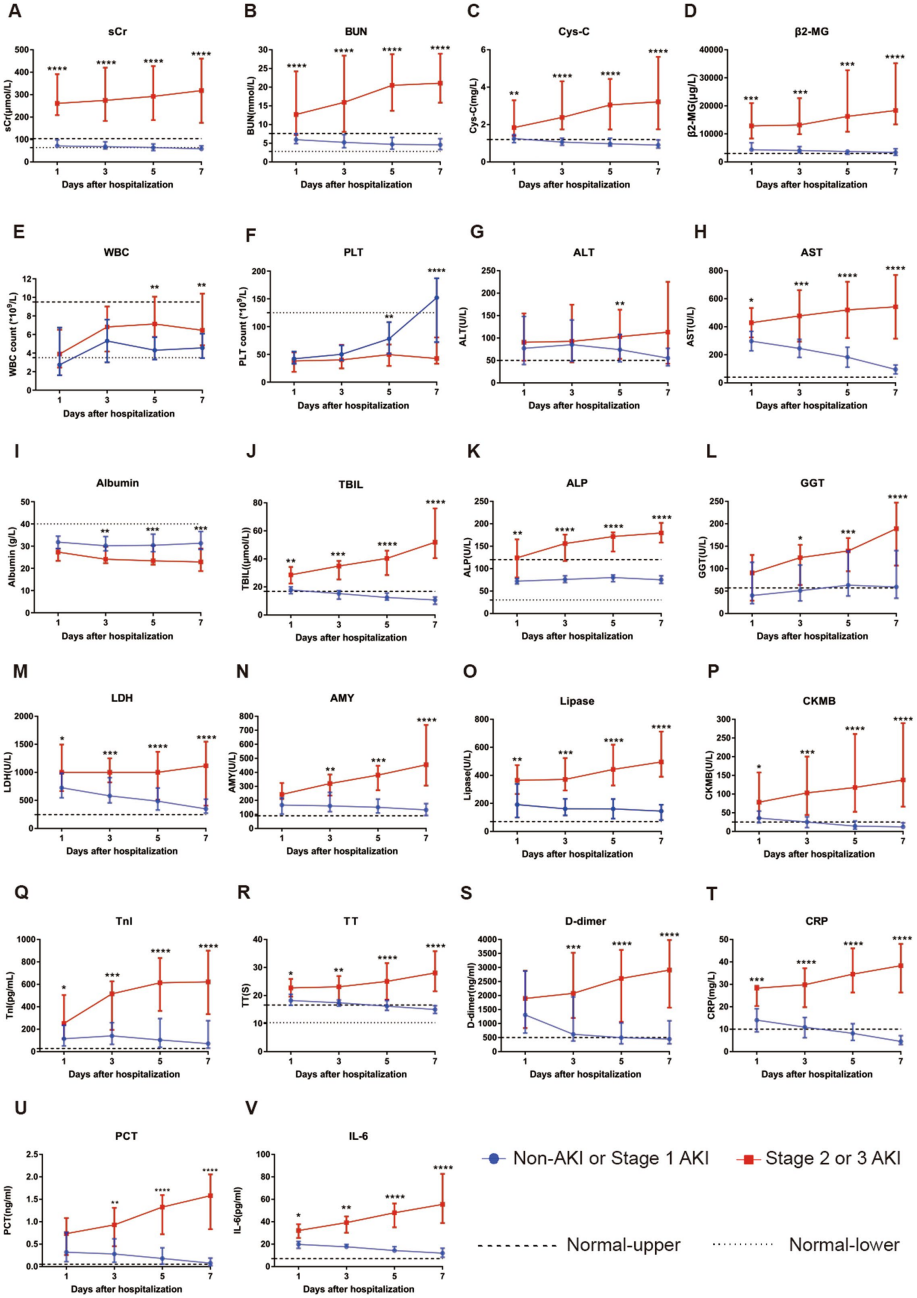
Evaluation of serial laboratory parameters for the non-AKI or stage 1 AKI group (*n* = 51) vs. stage 2 or 3 group (*n* = 16) of SFTS patients during hospitalization. **(A)** The longitudinal changes in sCr between the two groups. **(B)** The longitudinal changes in BUN between the two groups. **(C)** The longitudinal changes in Cys-C between the two groups. **(D)** The longitudinal changes in β2-MG between the two groups. **(E)** The longitudinal changes in WBC count between the two groups. **(F)** The longitudinal changes in PLT count between the two groups. **(G)** The longitudinal changes in ALT between the two groups. **(H)** The longitudinal changes in AST between the two groups. **(I)** The longitudinal changes in albumin between the two groups. **(J)** The longitudinal changes in TBIL between the two groups. **(K)** The longitudinal changes in ALP between the two groups. **(L)** The longitudinal changes in GGT between the two groups. **(M)** The longitudinal changes in LDH between the two groups. **(N)** The longitudinal changes in AMY between the two groups. **(O)** The longitudinal changes in lipase between the two groups. **(P)** The longitudinal changes in CKMB between the two groups. **(Q)** The longitudinal changes in TnI between the two groups. **(R)** The longitudinal changes in TT between the two groups. **(S)** The longitudinal changes in D-dimer between the two groups. **(T)** The longitudinal changes in CRP between the two groups. **(U)** The longitudinal changes in PCT between the two groups. **(V)** The longitudinal changes in IL-6 between the two groups. All data are presented as medians with interquartile ranges (P25–P75). ^*^p  <  0.05, ^**^p  <  0.01, ^***^p  <  0.001, and ^****^p  <  0.0001. Normal-upper: the upper limit of the normal value range. Normal-lower: the lower limit of the normal value range.

Subgroup patients with longitudinal laboratory test results were analyzed to investigate their serial changes with respect to disease severity. Levels of 22 laboratory parameters at four time points were collected and presented. Compared with patients without AKI or with stage 1 AKI, patients with stage 2 or 3 AKI showed a significant increase in serum concentrations of sCr, BUN, Cys-C, β2-MG, AST, TBIL, ALP, GGT, LDH, BUN, AMY, lipase, CK-MB, TnI, TT, D-dimer, CRP, IL-6, and PCT and significantly reduced serum levels of albumin and PLT count during the course of hospitalization (see Figure 3).

## Discussion

AKI is a clinical syndrome caused by a sudden decrease in renal excretory function, with the accumulation of nitrogen metabolism waste such as creatinine and urea. Other common clinical and laboratory presentations include reduced urine output, resulting in the accumulation of metabolic acids, potassium and phosphate. It is a frequent complication in hospital patients and very common in critically ill patients (Bellomo et al., 2012; Gonsalez et al., 2019). AKI in patients with SFTS was also observed in previous studies (Cui et al., 2015; Jia et al., 2017; Wang et al., 2019; He et al., 2021), yet with very little information on its incidence, clinical characteristics and prognosis.

To our knowledge, this is the first report of the clinical features and outcomes of AKI in SFTS. In the present study, we demonstrated the incidence of AKI in 26.4% of hospitalized SFTS patients. We also revealed that the patients with AKI showed higher decreased levels of albumin and fibrinogen, and elevated levels of AST, TBIL, ALP, LDH, BUN, uric acid, sCr, AMY, lipase, CKMB, TnI, APTT, TT and D-dimer than patients without AKI during hospitalization. Spearman correlation analysis also showed that sCr was closely correlated with these laboratory parameters. They are generally associated with fatal outcomes, and are commonly used to reflect the heart, liver, pancreas, kidney and coagulation functions, and their extremely abnormal levels reveal severe organ injury and dysfunction, which usually occur in critically ill patients, who should receive more attention, specifically by developing new therapeutic and prevention strategies to be used in clinical practice. Furthermore, we revealed that nonspecific symptoms including abdominal pain, diarrhea, encephalopathy and hepatosplenomegaly were more frequent in patients with AKI. Some studies have reported that encephalopathy and hepatosplenomegaly are independent predictors of mortality in patients with SFTS (Liu et al., 2013; Gong et al., 2021). It revealed that AKI was often linked with other severe complications, leading to poor prognosis.

A number of studies have revealed that a high viral load is generally associated with poor outcome. Which can be used to predict the mortality of patients with SFTS (Liu et al., 2016; Yang et al., 2016; Jo et al., 2022; Wang et al., 2022). We also found that the viral load was an independent risk factor for the prognosis of patients with SFTS. In the present study, the viral load was significantly higher in the AKI group than in the non-AKI group. At least in part, it could explain why patients in the AKI group have a higher mortality rate. Although the mechanisms of AKI in patients with SFTS are unclear, some studies reported that the SFTS virus was detected in the kidneys (Chen et al., 2012; Jin et al., 2012). Recently, a report showed that SFTS viral load in urine is an independent predictor of the incidence of AKI (Zhang et al., 2023). It implies that the SFTS virus may directly induce renal damage. Moreover, a large number of studies have shown that the SFTS virus can substantially produce inflammatory cytokines and chemokines, leading to cytokine storms (Khalil et al., 2021; Wang et al., 2021; Gao et al., 2022). It can promote kidney vascular permeability, and even microcirculatory dysfunction, and it can induce thrombosis and capillary leak syndrome, resulting in disseminated intravascular coagulation. In addition, these high serum levels of cytokines can directly cause the death of renal endothelial and tubular epithelial cells (Ahmadian et al., 2021; Legrand et al., 2021).

In the present study, we used the less sensitive sCr-based criteria to identify AKI. Although inulin clearance and radionuclide renal imaging are more sensitive, they are inconvenient and costly. Nonetheless, sCr measurement is still the most practical and widely accepted method for evaluating renal function in patients with AKI at present (Moledina et al., 2017; Moledina and Parikh, 2018). Some biomarkers of AKI including Cys-C and β2-MG were also evaluated in patients with SFTS. Cys-C is an elegant indicator of glomerular filtration rate because it is not affected by factors such as muscle mass, diet, drugs, sex and tubular secretion, and it is a reliable parameter for evaluating and predicting AKI in critically ill patients (Mårtensson et al., 2012; Luft, 2021). β2-MG has also traditionally been used to evaluate renal tubular function, and elevated β2-MG is typically accompanied by kidney injury (Bagshaw et al., 2007). Our results also showed that the serum levels of Cys-C and β2-MG in the AKI group were significantly higher than those in the non-AKI group.

Importantly, our findings confirmed that stage 2 or 3 AKI was a strong independent risk factor for the prognosis in patients with SFTS, and patients at AKI stage 2 or 3 showed a significantly higher in-hospital mortality than patients without AKI or at AKI stage 1. Some previous studies of other diseases also had yielded similar results. A large observational study demonstrated that the mortality of hospitalized patients at AKI stage 2 or 3 was approximately 2 or 4 times higher than that of patients at AKI stage 1, respectively (Khadzhynov et al., 2019). A retrospective analysis of the cohort of patients from the North American Consortium for the Study of End-Stage Liver Disease study on the complications of patients with decompensated cirrhosis revealed that patients at AKI stage 2 or 3 had higher model for end-stage liver disease scores, presence of systemic inflammatory response syndrome and second infections, resulting in a lower survival rate (Wong et al., 2021). Moreover, we compared the longitudinal changes in laboratory parameters of patients in the non-AKI, stage 1, 2 and 3 AKI groups. It revealed that laboratory indicators reflecting organ injury and inflammation returned to the normal range in patients without AKI or at AKI stage 1, but these parameters were aggravated continuously in patients at AKI stage 2 or 3 during hospitalization. These results could explain the higher mortality of patients at AKI stage 2 or 3.

Limitations of our study include the observational and retrospective study design. Additionally, the sample size of this study was relatively small. Therefore, the subsequent analysis of risk factors for AKI development in SFTS was not conducted.

At the same time, due to the limited laboratory resources in our hospital, some other AKI biomarkers including neutrophil gelatinase-associated lipocalin, kidney injury molecule-1 and interleukin-18 could not be measured. Finally, because this was a single-center study, the observations made here could not be extrapolated to other centers.

## Conclusion

In conclusion, we found a high incidence of AKI in patients with SFTS, and AKI was associated with poor prognosis. Most importantly, we revealed that patients at AKI stage 2 or 3 had higher mortality. These results provide strong evidence to emphasize that patients with SFTS should be carefully monitored for the development of AKI and corresponding measures should be taken to prevent it, and further explorations are required to identify the risk factors for the development of AKI.

## Data availability statement

The original contributions presented in the study are included in the article/Supplementary material, further inquiries can be directed to the corresponding authors.

## Ethics statement

The studies involving humans were approved by Ethics Committee of Zhongnan Hospital of Wuhan University. The studies were conducted in accordance with the local legislation and institutional requirements. The ethics committee/institutional review board waived the requirement of written informed consent for participation from the participants or the participants’ legal guardians/next of kin because the written informed consent was waived due to the nature of the retrospective study and pandemic nature of the disease.

## Author contributions

ZZ and XH were involved in patient data analysis and wrote the manuscript drafting. QJ and WH were mainly responsible for the data collection. AL, LD, and YX were responsible for the study design and critical revision. All authors contributed to the article and approved the submitted version.

## Funding

This work was supported by grants from Key Research and Development Program of Hubei Province, China (2020BCB025).

## Conflict of interest

The authors declare that the research was conducted in the absence of any commercial or financial relationships that could be construed as a potential conflict of interest.

## Publisher’s note

All claims expressed in this article are solely those of the authors and do not necessarily represent those of their affiliated organizations, or those of the publisher, the editors and the reviewers. Any product that may be evaluated in this article, or claim that may be made by its manufacturer, is not guaranteed or endorsed by the publisher.

## Figures and Tables

**Table:** 

AKI	acute kidney injury
ALT	alanine aminotransferase
AST	aspartate aminotransferase
ALP	alkaline phosphatase
AMY	amylase
APTT	activated partial thromboplastin time
BNP	brain natriuretic peptide
BUN	blood urea nitrogen
CK	creatinine kinase
CK-MB	creatinine kinase myocardial B fraction
CRP	C-reactive protein
Cys-C	cystatin C
ESR	erythrocyte sedimentation rate
GGT	gamma glutamyl transpeptidase
INR	international normalized ratio
IL-6	interleukin-6
LDH	lactate dehydrogenase
β2-MG	β2-microglobulin
OBT	occult blood test
PLT	platelet
PT	prothrombin time
PTA	prothrombin activity
PCT	procalcitonin
sCr	serum creatinine
SFTS	severe fever with thrombocytopenia syndrome
TBIL	total bilirubin
TC	total cholesterol
TG	triglyceride
TT	thrombin time
TnI	troponin I
UPT	urine protein test
UOBT	urine occult blood test
WBC	white blood cell
