# Supplementary material for: Clinical characteristics and outcomes of acute kidney injury in patients with severe fever with thrombocytopenia syndrome
Source: Front Microbiol. 2023 Sep 13;14:1236091. doi: 10.3389/fmicb.2023.1236091 (PMC10533938; doi:10.3389/fmicb.2023.1236091)
Supplement: Supplementary file 1 [file Data_Sheet_1.pdf]

Supplementary Figure S1: The study flow chart of the enrollment of patients

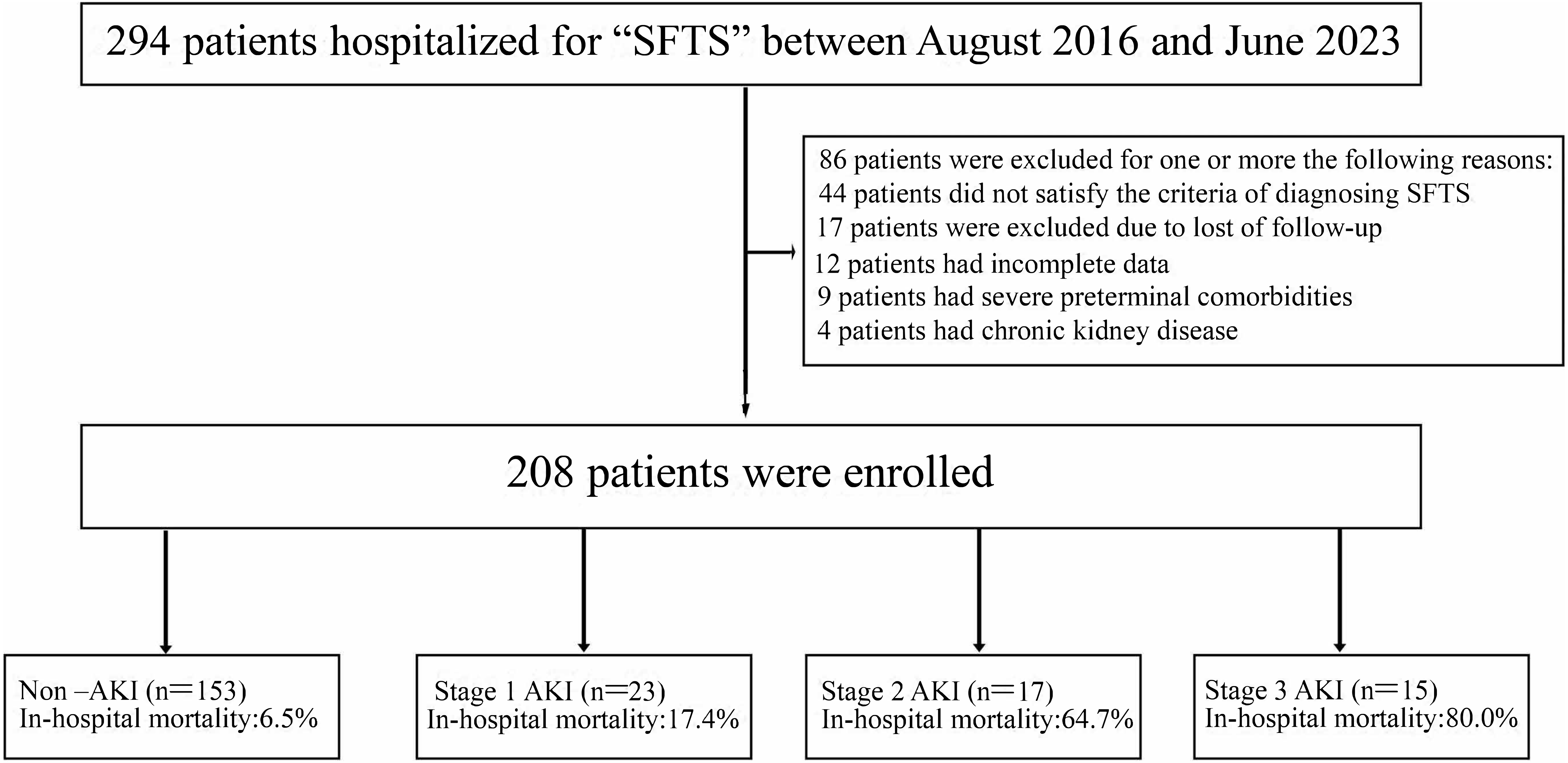

Supplementary Table S1. Comparison of demographics, comorbid conditions, clinical symptoms and laboratory parameters of SFTS patients at AKI stages 1, 2 and 3.

	Stage 1 AKI (n=23)	Stage 2 AKI (n=17)	Stage 3 AKI (n=15)
Male, n (%)	15(65.2)	7(41.2)	7(46.7)
Age (years)	65±6	69±8	67±7
Diabetes, n (%)	2(8.7)	3(17.6)	3(20.0)
Hypertention, n (%)	7(30.4)	5(29.4)	5(33.3)
Days from onset to admission	6(5-7)	8(7-9) *	8(7-10) &
Clinical manifestations, n (%)			
Fever >38 ℃	5(21.7)	5(29.4)	7(46.7) &
Headache	7(30.4)	4(23.5)	5(33.3)
Dizziness	9(39.1)	5(29.4)	5(33.3)
Cough	7(30.4)	5(29.4)	4(26.7)
Sputum	7(30.4)	2(11.8)	3(20.0)
Chest distress	5(21.7)	4(23.5)	5(33.3)
Anorexia	21(91.3)	13(76.5)	13(86.7)
Nausea	17(73.9)	13(76.5)	14(93.3)
Vomiting	4(17.4)	3(17.6)	5(33.3)
Abdominal pain	6(26.1)	9(52.9)	6(40.0)
Diarrhea	2(8.7)	5(29.4)	6(40.0)
Petechia	2(8.7)	4(23.5)	6(40.0)
Encephalopathy	3(13.0)	6(35.3)	9(60.0) &
Hepatosplenomegaly	2(8.7)	4(23.5)	5(33.3)
Laboratory parameters			
WBC (10^9/L)	5.22(2.50-9.23)	2.80(2.44-4.32) **	5.14(4.16-6.59)
Neutrophils (%)	75.3(63.1-85.7)	70.9(61.0-80.1)	71.7(59.0-84.7)
Neutrophils (10^9 /L)	4.1(1.4-6.4)	1.9(1.3-3.5) **	4.1(2.5-5.8)
Lymphocyte (%)	14.9(11.2-30.0)	24.5(13.4-30.1)	21.2(10.7-23.7)
Lymphocyte (10^9/L)	0.8(0.5-1.2)	0.6(0.5-0.9)	0.9(0.6-1.5)
Hemoglobin (g/L)	117±15	120±21	123±20
PLT (10^9 /L)	45(37-63)	36(26-58)	33(22-50) &
ALT (U/L)	68(47-193)	93(52-243) *	165(91-288) && #
AST (U/L)	200(115-508)	422(271-1068) **	624(376-1271) &&& #
TBIL(μmol/L)	12.6(8.2-17.7)	13.8(9.7-18.3)	15.0(11.6-29.2) &
DBIL(μmol/L)	5.0(3.3-10.0)	5.6(4.1-11.4)	8.5(4.8-20.7) &
Albumin (g/L)	28.4±4.0	26.4±6.6	25.1±3.6
ALP (U/L)	93(41-142)	106(57-164)	110(66-275) &
GGT (U/L)	89(32-104)	96(48-152)	110(68-325) &
LDH (U/L)	829(581-997)	942(680-1385) *	1082(831-3239) && #
TC (mmol/L)	3.22(2.38-3.73)	3.19(2.64-4.21)	2.77(1.99-3.69)
TG (mmol/L)	2.31(1.81-3.08)	2.95(2.17-3.68)	2.81(2.29-4.70)
BUN (mmol/L)	7.3(4.8-11.2)	8.0(5.2-15.6)	16.7(10.6-25.2) &&
Uric acid (μmol/L)	399(368-489)	450(413-504) *	560(464-580) &

sCr (μmol/L)	144(133-154)	205(179-231) **	367(307-418) &&& #
Cys-C (mg/L)	1.73(1.38-2.21)	1.92(1.86-2.73) *	3.56(2.32-4.20) &&& #
β2-MG (μg/L)	4138(3752-5278)	4852(4059-8234) *	7729(6946-15348) && #
Sodium (mmol/L)	136(133-140)	135(129-139)	136(131-142)
Potassium (mmol/L)	3.9(3.6-4.2)	4.2(3.3-4.5)	4.4(3.8-5.1)
AMY (U/L)	170(106-228)	203(137-365) *	260(180-342) && #
Lipase (U/L)	210(55-464)	272(128-576) *	322(235-680) &
CK (U/L)	305(226-1253)	396(435-1628)	703(679-2083) &
CK-MB (U/L)	28(18-52)	41(37-89) *	112(63-301) && #
TnI (pg/mL)	161.3(50.7-276.5)	217.0(117.7-563.5) **	324.3(202.4-619.6) &&& #
BNP (pg/mL)	82.3(42.5-108.4)	120.8(95.2-238.4) *	165.0(138.5-295.4) && #
PT (s)	11.5(11.2-11.7)	11.9(11.5-12.6)	12.1(11.7-13.8)
INR	1.04(1.02-1.07)	1.09(1.03-1.15)	1.11(1.06-1.27)
APTT(s)	39.8(35.4-44.9)	41.6(36.1-50.3)	54.2(47.0-65.7) &
TT(s)	18.0(16.2-20.4)	18.3(16.6-22.3)	22.8(20.7-29.9) &
Fibrinogen(mg/dL)	254(210-288)	147(135-214)	227(148-273)
D-dimer (ng/mL)	1069(452-2254)	1352(696-2713) *	1958(934-3678) && #
CRP (mg/L)	6.8(2.5-16.9)	8.4(4.1-18.5) *	11.0(7.1-28.7) && #
PCT (ng/mL)	0.24(0.04-0.67)	0.38(0.18-0.95) *	1.46(0.52-2.57) && #
IL-6 (pg/mL)	30.6(14.7-106.3)	40.6(25.3-151.2) *	174.9(93.4-583.5) && #
ESR (mm/h)	7(4-13)	8(6-17) *	12(7-29) && #
Viral load (log10 copies/ml)	4.0(3.1-4.9)	4.3(3.8-6.1) *	5.9(5.0-7.4) && #
OBT positive, n (%)	7(30.4)	5(29.4)	6(40.0)
UPT positive, n (%)	19(82.6)	15(88.2)	12(80.0)
UOBT positive, n (%)	18(78.3)	15(88.2)	12(80.0)
Urine RBC count	24.6(10.3-85.2)	55.3(27.6-145.2) *	93.5(48.1-317.5) && #

Note: **P* value<0.05, ** *P* value<0.01 for comparisons between SFTS patients at AKI stage 1 and at AKI stage 2. & *P* value<0.05, && *P* value<0.01, &&& *P* value<0.001 for comparisons between SFTS patients at AKI stage 1 and at AKI stage 3. # *P* value<0.05 comparisons between SFTS patients at AKI stage 2 and at AKI stage 3.

Supplementary Table S2. Comparison of demographics, comorbid conditions, clinical symptoms and laboratory parameters of SFTS patients in the survival and fatal groups.

	All (n=208)	Survival (n=171)	Fatal (n=37)	*P* value
Male, n (%)	110(52.9)	88(51.5)	22(59.5)	0.377
Age (years)	65±8	64±8	67±7	0.055
Diabetes, n (%)	15(7.2)	10(5.8)	5(13.5)	0.102
Hypertention, n (%)	53(25.5)	46(26.9)	7(18.9)	0.312
Days from onset to admission	7(6-9)	6(5-7)	7(5-8)	0.342
Clinical manifestations, n (%)				
Fever >38 ℃	50(24.0)	34(19.9)	16(43.2)	0.003
Headache	40(19.2)	29(17.0)	11(29.7)	0.074
Dizziness	62(29.8)	48(28.0)	14(37.8)	0.239
Cough	55(26.4)	45(26.3)	10(27.0)	0.929
Sputum	41(19.7)	34(19.9)	7(18.9)	0.894
Chest distress	42(20.2)	33(19.3)	9(24.3)	0.490
Anorexia	153(73.6)	122(70.4)	31(81.8)	0.120
Nausea	160(76.9)	130(76.0)	30(81.0)	0.331
Vomiting	53(25.5)	43(25.1)	10(27.0)	0.812
Abdominal pain	55(26.4)	43(25.1)	12(32.4)	0.362
Diarrhea	33(15.9)	26(15.2)	7(18.9)	0.575
Petechia	22(10.6)	14(8.2)	8(21.6)	0.016
Encephalopathy	32(15.4)	16(9.4)	16(43.2)	<0.001
Hepatosplenomegaly	23(11.1)	13(7.6)	10(27.0)	0.001
Stage 2 or 3 AKI	32(15.4)	8(4.7)	24(64.9)	<0.001
Laboratory parameters				
WBC (10^9/L)	3.81(2.30-6.40)	3.5(2.1-6.1)	4.9(3.2-6.9)	0.052
Neutrophils (%)	69.8(55.5-82.1)	69.7(52.4-82.0)	69.9(57.9-82.9)	0.750
Neutrophils (10^9/L)	2.3(1.2-5.1)	1.9(1.1-4.9)	3.4(2.0-5.6)	0.088
Lymphocyte (%)	21.1(11.9-30.8)	20.6(11.6-31.1)	21.1(13.3-27.5)	0.747
Lymphocyte (10^9/L)	0.7(0.5-1.1)	0.7(0.5-1.0)	0.8(0.5-1.4)	0.286
Hemoglobin (g/L)	122±23	124±22	116±29	0.110
PLT (10^9 /L)	41(29-58)	42(31-58)	34(22-59)	0.137
ALT (U/L)	79(46-145)	69(45-115)	142(80-245)	<0.001
AST (U/L)	244(106-496)	172(94-336)	666(335-1246)	<0.001
TBIL (μmol/L)	10.9(8.2-15.6)	10.7(8.1-14.6)	13.1(9.3-22.7)	0.092
DBIL (μmol/L)	4.5(2.8-8.2)	4.1(2.7-6.4)	7.7(4.8-14.4)	<0.001
Albumin (g/L)	29.3±5.0	29.8±5.1	27.3±4.1	0.005
ALP (U/L)	42(26-104)	70(54-90)	110(68-195)	0.001
GGT (U/L)	25(23-29)	37(23-96)	91(37-238)	<0.001
LDH (U/L)	782(481-1000)	684(446-938)	1000(1000-2482)	<0.001
TC (mmol/L)	2.88(2.42-3.55)	2.93(2.41-3.60)	2.80(2.13-3.18)	0.194
TG (mmol/L)	2.23(1.55-2.99)	2.17(1.53-2.88)	2.65(1.85-3.82)	0.114

BUN (mmol/L)	5.6(4.1-8.4)	5.3(4.0-7.3)	8.2(5.2-13.6)	<0.001
Uric acid (μmol/L)	305(243-397)	291(235-370)	433(319-493)	<0.001
sCr (μmol/L)	79(65-138)	74(64-101)	210(80-339)	<0.001
Cys-C (mg/L)	1.26(1.01-1.58)	1.21(0.98-1.47)	1.52(1.19-3.31)	0.001
β2-MG (μg/L)	4067(3422-5410)	3844(3409-4714)	5418(4157-3226)	0.064
Sodium (mmol/L)	135(132-138)	135(132-137)	134(130-139)	0.681
Potassium (mmol/L)	3.7(3.3-4.2)	3.6(3.3-4.0)	4.1(3.5-4.8)	0.001
AMY (U/L)	160(99-233)	154(92-205)	260(139-347)	<0.001
Lipase (U/L)	183(93-358)	164(76-283)	322(154-548)	<0.001
CK (U/L)	485(196-1367)	437(135-1238)	1184(479-2583)	0.001
CK-MB (U/L)	37(17-60)	32(15-47)	77(40-177)	<0.001
TnI (pg/mL)	122.5(46.4-262.6)	106.6(41.0-231.4)	215.3(102.8-587.0)	0.003
BNP (pg/mL)	78.0(128.3-192.5)	72.5(56.3-108.4)	165.0(138.5-295.4)	0.275
PT (s)	11.4(10.8-12.3)	11.3(10.7-12.0)	11.9(11.2-12.8)	0.008
INR	1.04(0.99-1.13)	1.04(0.98-1.10)	1.09(1.03-1.17)	0.008
PTA (%)	97(88-108)	98(89-109)	94(81-104)	0.105
APTT (s)	41.6(36.1-50.3)	39.8(35.4-44.9)	54.2(47.0-65.7)	<0.001
TT (s)	18.3(16.6-22.3)	18.0(16.2-20.4)	22.8(20.7-29.9)	<0.001
Fibrinogen (mg/dL)	234(183-290)	251(200-297)	165(143-234)	<0.001
D-dimer (ng/mL)	1162(483-2558)	1039(397-2014)	1922(885-3473)	0.011
CRP (mg/L)	7.1(2.4-17.5)	4.7(1.5-14.9)	11.0(7.1-28.7)	0.004
PCT (ng/mL)	0.26(0.08-0.83)	0.18(0.07-0.56)	1.14(0.30-2.18)	<0.001
IL-6 (pg/mL)	41.0(15.1-115.3)	30.6(13.3-81.6)	167.0(80.9-569.0)	<0.001
ESR (mm/h)	8（4-14）	8(4-14)	9(5-27）	0.381
Viral load (log_{10} copies/ml)	4.2(3.2-5.4)	3.7(2.8-4.5)	5.7(4.8-7.1)	<0.001
OBT positive, n (%)	49(23.6)	38(22.4)	11(30.3)	0.345
UPT positive, n (%)	171(82.2)	140(81.9)	31(83.8)	0.252
UOBT positive, n (%)	163(78.4)	132(77.2)	31(83.8)	0.833
Urine RBC count	20.2(8.8-70.4)	15.9(7.6-39.2)	80.5(29.3-294.2)	<0.001
